# The effect of four different intracanal medicaments on the push-out bond strength of root canal sealers

**DOI:** 10.25122/jml-2020-0104

**Published:** 2022-04

**Authors:** Shalu Maan, Vijaya Dhar Bhatt, Rohit Singh, Sayak Gupta, Syed Alay Noorain, Aashna Gill, Pradeep Kumar, Sushil Yadav, Preeti Sharma

**Affiliations:** 1. Department of Conservative Dentistry and Endodontics, Santosh Dental College, Santosh Deemed to be University, Ghaziabad, India; 2. Department of Ophthalmology, Lala Lajpat Rai Memorial Medical College, Meerut, Uttar Pradesh, India; 3. Department of Biochemistry, Amar Shahid Jodha Singh Ataiya Thakur Dariyav Singh Medical College, Fatehpur, Uttar Pradesh, India; 4. Department of Biochemistry, Santosh Medical College, Santosh Deemed to be University, Ghaziabad, India

**Keywords:** AH Plus, sealing effect, MTA Fillapex push-out bond strength

## Abstract

This study aimed to compare the effect of 4 different intracanal medicaments on the push-out bond strength of two sealers (AH Plus and MTA Fillapex). 100 single-rooted extracted premolar teeth were divided into 5 groups, with 20 samples in each group, one group being the control group. After cleaning and shaping procedures, the canals were filled with 4 different medicaments: calcium hydroxide, tri-antibiotic paste (TAP), Metapex, or Chlorhexidine (2%) gel for 2 weeks. Following this, the medicaments were rinsed away, and the samples in those sub-groups were obturated with gutta-percha/AH Plus or MTA Fillapex sealers. After 2 weeks of incubation, a 2-mm-thick middle section from each root was evaluated to test push-out strength. The obtained data were tabulated, and appropriate statistical analysis was performed (two-way ANOVA and LSD test). When comparing the average values, the bond strength values of AH Plus were significantly higher than those of MTA Fillapex (p<0.05) in all medicament groups. Based on the findings, we concluded that AH Plus had comparatively higher bond strength than MTA Fillapex. We also observed that AH Plus had higher bond strength in the presence of calcium hydroxide, whereas MTA Fillapex in the presence of Chlorhexidine. A comparison of the push-out bond strength shows that irrespective of the root canal segment or the final irrigant used, AH Plus showed higher values among all groups. The limitation of the current study was that the effect of TAP on the bond strength of endodontic sealers was not negative.

## Introduction

The concurrence between root canal wall and root canal filling material is settled by various endodontic sealers. A durable and efficient attachment of the filling material is essential to prevent root canal infections generally caused by the growth of microbes and newer infections caused by coronal apical leakage [1]. When evaluating the properties of different sealers, the most important feature is that the root canal environment is prepared with a bacteria-tight seal, which is maximally attained by the endodontic sealer. Dislodgement resistance, also known as the push-out bond strength (POBS), is considered a significant prognostic marker to determine the compatibility between the root canal wall, sealer, and core material [1]. It is evident that adherence to dentin is a fundamental feature of root canal sealers; studies and tests have proven that a stronger bond strength diminishes the leaking of the canal and improves the stability of root canal obturation material. The extent to which the sealer adheres to dentin is determined by factors such as variations in the structure of root dentin amongst the inter-samples or intra-sample, presence/absence of smear layer, chemical constituents of sealer, and its interaction with the dentin [2]. Gutta-percha (GP) is incapable of independently providing a complete seal; therefore, without a sealer, it is almost impossible to acquire a complete seal of the root canal [2]. Recently, a superior sealer was developed, namely mineral trioxide aggregate (MTA), which was shown to be biocompatible and bactericidal. This sealer shows a positive effect on the smear layer removal, and it has high push-out bond strength that helps prevent material dislodgement [3]. Sealers also play a critical role in the retention of the post, which can be affected during cementation; the type of sealer used can interfere with the bond strength between sealer and root canal dentin [4]. Various techniques were proposed to control the spread of micro-organisms in the root canal system, including instrumentation techniques, irrigation protocols, and intracanal medicaments.

Therefore, to reduce the bacterial count within the root canals, several intra-canal dressings were recommended by researchers [5]. Over the years, studies showed that intracanal medicaments have multiple clinical applications, including managing traumatized teeth, treating periapical lesions, revascularization of immature teeth, apexification, or inflammatory root resorption [6]. Calcium hydroxide is more frequently used as an intracanal medicament due to its high efficacy against bacteria and low cytotoxicity. Calcium hydroxide in the aqueous environment causes the release of Hydroxyl, leading to bacterial cytoplasmic membrane damage and protein denaturation, affecting the DNA strand of the bacteria [6]. Metapex is a new root canal filling material containing calcium hydroxide, iodoform, and silicone oil media. The efficacy of Metapex against bacterial activity in the root canal is superior in comparison to plain calcium hydroxide [7]. In the present study, Metapex was chosen as a test material given its chemical composition compared to plain calcium hydroxide on the bond strength of sealers and the dentin [7]. A combination of anti-bacterial triple antibiotic paste (TAP) was introduced to treat, regenerate, and revascularize necrotic pulp with an open apex. TAP consists of ciprofloxacin, metronidazole, minocycline [8]. Metronidazole shows an anti-microbial effect against protozoa, being toxic to anaerobes. Minocycline has a bacteriostatic effect on gram-negative and gram-positive bacteria, while ciprofloxacin, a synthetic fluoroquinolone, shows bactericidal action against gram-negative bacteria with limited activity against gram-positive bacteria. This combination helps destroy odontogenic micro-organisms. TAP is a good material of choice [8]. Chlorhexidine plays a pivotal role as an anti-microbial agent during disinfection of root canal spaces, orifice widening and removing necrotic tissue, chemo-mechanical preparation, and prior to patency and canal enlargement. It can be used as intracanal medicament alone or combined with other substances like calcium hydroxide in disinfection obturation cones to remove gutta-percha cones during retreatment.

The gel form of Chlorhexidine is available, consisting of natrosol (hydroxyethylcellulose) gel and chlorhexidine gluconate in optimal pH range 5.5 to 7.0. Therefore, it can be easily removed from the root with the help of distilled water. Compared to the liquid form, Chlorhexidine gel is viscous and helps reduce smear layer formation by keeping dentinal debris in suspension [9]. So far, no study can assess the effects of other intracanal medicaments, like triple antibiotic paste and other compositions of calcium hydroxide such as Metapex and 2% CHX on the bond strength of MTA Fillapex and AH Plus [7]. The objective of this study is to evaluate this.

## Material And Methods

This study included 100 caries-free samples, non–restored freshly extracted permanent single-rooted premolar teeth (initial apical, file size not more than no.15) taken from the Oral and Maxillofacial Surgery Department, Santosh Dental College and Hospital, Ghaziabad. Teeth roots were assessed for any pathology such as open apices, caries, restorations, and cracks, and teeth with any of these conditions were excluded from the study. Chloramines solution (0.5%) was used for storing the specimens. A diamond disk was used to remove the crown of the tooth. A root length of 15 mm was achieved in those samples. The working length (WL) was determined using the #15 file. The file was introduced in the root canal, and 0.5 short of the measurement, once the file tip was visible under a magnifying glass at the apical foramen was considered WL. Next, the crown down technique was used to prepare the root canals for the F3 universal protaper. A 27 gauge syringe was used for root canal irrigation, and 2 ml of Naocl (5.2%) were used for this purpose, followed by 5 ml of EDTA (17%). Finally, the rinsing was done using 5 ml of distilled water. Paper points were used for the drying of root canals.

A set of 5 groups, each comprising of 20 samples, was divided as follows:

• Group 1: control group: no medicaments placed, following cleaning and shaping, teeth were obturated;
• Group 2: a pure mix of Ca(OH)_2_ placed in the root canal using recommendations provided by the manufacturer;
• Group 3: samples injected with Metapex into the root canals as per the recommendations of the manufacturer;
• Group 4: samples injected with a mixture of metronidazole, ciprofloxacin, and minocycline (250 mg each) in distilled water;
• Group 5: a 2% CHX was injected into the root canals using a special syringe provided by the manufacturer.

The samples were incubated at 37°C for 2 weeks. Later on, the intracanal medicaments were removed using distilled water with the help of a #40k file.

• The two sub-groups were divided as follows:
• Subgroup A: This sub-group comprised samples obturated with gutta-percha points and AH Plus sealer.
• Subgroup B: This sub-group comprised samples obturated with gutta-percha and MTA Fillapex.

Post obturation x-ray was taken to confirm the proper setting. All samples were incubated at 37°C after sealing with Cavit for 2 weeks. Root samples were then sectioned into 2 mm cross-sections from the middle third of the perpendicular to the root surface with the help of a disk (Buehler). Twenty slices were obtained in each of the groups.

A force was applied on the sample surface using a cylindrical piston of 0.8 mm diameter at a 1 mm/min crosshead speed perpendicular to the sample surface with the help of a universal testing machine. The force applied for material displacement was noted in newton and changed to MPa using a formula as such:

Dentin thickness in the slice x the canal circumference = the surface area under load.

The effect of medicaments on push-out bond strength was assessed by two-way ANOVA. In addition, a post hoc LSD test was also used to precisely evaluate the differences between the medicaments. A p-value <0.05 was considered to be statistically significant.

## Results

We identified that the overall mean bond strength of AH Plus was significantly higher than MTA Fillapex.

We compared the bond strength between MTA Fillapex and AH Plus groups using an unpaired t-test, as shown in Table 1. The average bond strength was significantly higher in the AH Plus subgroups than in the MTA Fillapex subgroups.

**Table 1.** Comparison of mean bond strength between MTA Fillapex and AH plus groups

	**MTA Fillapex**		**AH Plus**		**Mean Difference**	**p-value**
	**Mean**	**Std. Deviation**	**Mean**	**Std. Deviation**		
**TAP**	1.27	0.36	3.38	0.50	-2.11	<0.001*
**CHX**	1.96	0.69	4.06	0.62	-2.10	0.001*
**Metapex**	1.64	0.78	4.18	0.71	-2.54	0.001*
**Calcium hydroxide**	2.01	0.12	4.95	0.89	-2.94	<0.001*
**Control**	0.81	0.22	1.65	0.65	-0.83	0.027*

Unpaired t-test. * – Significant difference.

We compared the mean bond strength between MTA Fillapex and AH Plus groups using an unpaired t-test (Table 2). The mean bond strength was significantly higher in the AH Plus group than in the MTA Fillapex group.

**Table 2.** Comparison of mean bond strength between MTA Fillapex and AH plus groups.

	**Mean**	**Std. Deviation**	**Mean Difference**	**p-value**
**MTA Fillapex**	1.54	0.65	-2.10	<0.001*
**AH Plus**	3.64	1.30		

Unpaired t-test. * – Significant difference.

We compared the mean bond strength between TAP, CHX, Metapex, calcium hydroxide, and control groups using the one-way ANOVA test (Table 3). There was a significant difference in mean bond strength between TAP, CHX, Metapex, calcium hydroxide, and control groups.

**Table 3.** Comparison of mean bond strength between TAP, CHX, Metapex, calcium hydroxide, and control groups.

	**Mean**	**Std. Deviation**	**p-value**
**TAP**	2.32	1.19	0.004*
**CHX**	3.01	1.27	
**Metapex**	2.91	1.51	
**Calcium hydroxide**	3.48	1.66	
**Control**	1.23	0.64	

Unpaired t-test. * – Significant difference.

We compared the inter-group mean bond strength using the post hoc Bonferroni test (Table 4). The mean bond strength was significantly higher among TAP, CHX, Metapex, and calcium hydroxide than the control group. The mean bond strength was significantly higher among calcium hydroxide than TAP.

**Table 4.** Inter-group comparison of mean bond strength between all groups.

**Groups**	**Groups**	**Mean Difference**	**p-value**
**TAP**	CHX	-0.69	1.000
**TAP**	Metapex	-0.59	1.000
**TAP**	Calcium hydroxide	-1.16	0.048*
**TAP**	Control	1.10	0.042*
**CHX**	Metapex	0.10	1.000
**CHX**	Calcium hydroxide	-0.47	1.000
**CHX**	Control	1.78	0.037
**Metapex**	Calcium hydroxide	-0.57	1.000
**Metapex**	Control	1.68	0.038*
**Calcium hydroxide**	Control	2.25	0.003*

Post-hoc Bonferroni test. * – Significant difference.

**Table 5.** Comparison of mean bond strength between TAP, CHX, Metapex, Calcium hydroxide, and control groups.

**MTA Fillapex**	**Mean**	**Std. Deviation**	**p-value**
**TAP**	1.27	0.36	0.006*
**CHX**	1.96	0.69	
**Metapex**	1.64	0.78	
**Calcium hydroxide**	2.01	0.12	
**Control**	0.81	0.22	

One-way Anova test. * – Significant difference.

**Table 6.** Inter-group comparison of mean bond strength between all the groups.

**Groups**	**Groups**	**Mean Difference**	**p-value**
**TAP**	CHX	-0.69	0.425
**TAP**	Metapex	-0.38	1.000
**TAP**	Calcium hydroxide	-0.74	0.034*
**TAP**	Control	0.46	0.040*
**CHX**	Metapex	0.32	1.000
**CHX**	Calcium hydroxide	-0.05	1.000
**CHX**	Control	1.15	0.018*
**Metapex**	Calcium hydroxide	-0.37	1.000
**Metapex**	Control	0.83	0.169
**Calcium hydroxide**	Control	1.20	0.012*

Post-hoc Bonferroni test. * – Significant difference.

**Table 7.** Comparison of mean bond strength between all groups.

**AH Plus**	**Mean**	**Std. Deviation**	**p-value**
**TAP**	3.38	0.50	<0.001*
**CHX**	4.06	0.62	
**Metapex**	4.18	0.71	
**Calcium hydroxide**	4.95	0.89	
**Control**	1.65	0.65	

One-way ANOVA test. * – Significant difference.

**Table 8.** Inter-group comparison between TAP, CHX, Metapex, Calcium hydroxide, and control group.

**Groups**	**Groups**	**Mean Difference**	**p-value**
**TAP**	CHX	-0.68	1.000
**TAP**	Metapex	-0.80	0.804
**TAP**	Calcium hydroxide	-1.57	0.017*
**TAP**	Control	1.73	0.007*
**CHX**	Metapex	-0.12	1.000
**CHX**	Calcium hydroxide	-0.89	0.538
**CHX**	Control	2.41	<0.001*
**Metapex**	Calcium hydroxide	-0.77	0.915
**Metapex**	Control	2.53	<0.001*
**Calcium hydroxide**	Control	3.30	<0.001*

Post-hoc bonferroni test. * – Significant difference.

We compared the mean bond strength between TAP, CHX, Metapex, calcium hydroxide, and control groups using the one-way ANOVA (Table 5). There was a significant difference in mean bond strength between TAP, CHX, Metapex, calcium hydroxide, and control groups.

The inter-group comparison of mean bond strength was performed using the post hoc Bonferroni test. The mean bond strength was significantly higher among TAP, CHX, Metapex, and calcium hydroxide than the control group. The mean bond strength was significantly higher in the calcium hydroxide than the TAP group (Table 6).

We compared the mean bond strength between TAP, CHX, Metapex, calcium hydroxide, and control groups using the one-way ANOVA test. There was a significant difference in mean bond strength between TAP, CHX, Metapex, calcium hydroxide, and control groups (Table 7).

An inter-group comparison of mean bond strength was performed using the post hoc Bonferroni test. The mean bond strength was significantly higher among TAP, CHX, Metapex, and calcium hydroxide than the control group. It is seen in Table 8 that the mean bond strength was significantly higher among calcium hydroxide than TAP.

## Discussion

The analysis of push-out bond strength is a methodical approach to investigate the dislodgement aversion of a sealer. The strength of the sealer to the core material or the root canal wall is tested considering the effect of a sealer, within the constraints of the push-out bond strength analysis, with root dentin being one of the most important aspects for the success of endodontic phenomenon [1]. The procedure is widely accepted and thus has been used in this study to record the interfacial bond strength of filling the root canal materials and is also a technique for radicular dentin at the lowest level [10]. The multiple test panels for measuring the bond strength are push-out, shear, and micro tensile strength tests. A study by Goracci *et al.* concluded that the push-out test is very reliable as this method gives a clear clinical picture in determining the binding efficiency of the root canal sealer [11]. Therefore, we chose this method in our study. This study used two sealers, AH Plus and MTA Fillapex, with various intracanal medicaments and compared their bond strengths. We found that the bond strength of AH Plus was higher than that of MTA Fillapex. The average bond strength was significantly higher among calcium hydroxide, Metapex, TAP, and CHX than in the control group. Adhesion to root dentin is the critical property of root canal sealers for two important reasons: (1) there is decreased apical and coronal leakage by superior, and (2) the filling material is fixed and stops its dislocation during procedures. The chemical composition of sealers is the major factor in determining their adhesion characteristics [12, 13].

ZOE-based sealers are the conventional sealers used by dentists worldwide. The eugenol portion of these sealers is chelated by zinc oxide present in gutta-percha. Inappropriate polymerization of these sealers can cause stress on root canal walls, resulting in microleakage, marginal gaps, and a few clinical failures [14]. One side of the root canal may detach due to forces of polymerization shrinkage which may exceed its bond strength to root dentin, and a strong bond with the root canal dentin cannot be formed [15]. Nevertheless, some sealers are present on the market, like Methacrylate-based resin sealers. These sealers help attach the filling material and root dentin. It penetrates the resin tags inside the dentinal tubules and gets adhered to the collagen matrix to seal the root canal effectively [2]. In the current study, it was evident that the AH Plus sealer showed stronger adherence to radicular dentin than the other sealer. Consequently, AH Plus sealer was considered to control strata in earlier studies since it is a gold standard among the sealers. The epoxy resins present in these resin-based sealers as a constituent is responsible for the tough bond strength of these sealers, as reported by Cecchin *et al.* [4]. When AH Plus and MTA Fillapex are evaluated through SEM images, greater magnification can be highly significant to observe the sealer adaptation and penetration into the dentine tubules as push-out strength can be studied in detail. The present study shows that failure modes were seen in both sealers, AH Plus sealer, and mixed pattern for MTA Fillapex. A tough bond between radicular dentin and AH Plus ensures that the push-out test values are represented by the cohesive failure.

The penetration of the sealer in dentine tubules and the formation of the tag were generally observed. We identified that the dentine tubules were observable even in the adhesion failure area. Even though the orifices were open, no sealer penetrated the dentinal tubules efficiently. Such patterns were previously reported for other sealers [13]. Various literature concluded that AH Plus sealer has comparatively higher bond strength than MTA Fillapex. However, few studies state that these sealers are equally strong enough and resist similarly against dislodgement. A considerable difference in setting time was seen among these sealers [3].

Mandava *et al.* evaluated and compared the effect of two resin sealers, AH Plus and MetaSEAL, and an MTA sealer MTA Fillapex on endodontically treated teeth for resistance to fractures. They concluded that AH Plus sealer is more effective in avoiding fractures among the various sealer groups. There was no significant difference among MetaSEAL and MTA Fillapex groups regarding fracture resistivity [12]. One of the intracanal medicaments used in the present study is TAP, an amalgamation of triple antibiotics, which is usually applied to treat the necrotic pulp of open apex teeth and promote teeth revascularization and regeneration. It has numerous other uses in endodontics [8]. As intracanal medicaments, calcium hydroxide was applied by Herman in 1920 as a direct pulp capping agent in endodontics. Generally, the presence of numerous residual bacteria causes endodontic failures, which can be easily prevented by applying an inter-appointment medicament in the canal, and for this purpose, calcium hydroxide is frequently used. It requires 7 days for efficient disinfection. Our study recorded the highest push-out bond strength when calcium hydroxide was used as an intracanal medicament [13]. Other researchers had similar findings and stated that calcium hydroxide Ca(OH)_2_ used as a treatment for 14 days showed satisfying effects on the bond strength of AH Plus to root dentin [14–18]. However, according to Amin *et al.*, intracanal treatment with Ca(OH)_2_ for a week showed no changes in the bond strength of AH Plus. Uzun *et al.* claimed that significantly higher push-bond strength was observed in AH Plus sealer in the apical root canal after using natural antibiotic propolis for intracanal treatment than treatment with Ca(OH)_2_ [17]. At the same time, we found the highest bond strength with calcium hydroxide only in the middle section of the root. The last and most effective intracanal medicament against E.fecalis and C.albicans used in the present study is Chlorhexidine (CHX). As shown by other studies, CHX liquid was found to be equally or slightly more effective than CHX gel. In other studies, 2% CHX liquid was seen to have lesser anti-microbial properties than the gel form of 2% CHX. Francisco *et al.* explained that CHX gel has many more advantages than CHX solution, although they are similar in many aspects such as anti-microbial, biocompatibility, and substantivity properties. When the root canal walls are lubricated by CHX gel, it helps minimize the friction effect between the dentin surface and file. This lubrication also facilitates instrumentation and minimizes the serious risks of the breakage of instruments inside the canal while doing the procedures.

Moreover, by facilitating instrumentation, the organic tissues can be eliminated easily by CHX gel [9]. In a study by Gupta *et al.*, AH Plus was the outstanding performer, exhibiting the maximum push-out bond strength values in the coronal one-third of the root when CHX was used as the final irrigant. The major reason for high push-out bond strength could be that CHX enhances the bond strength of resin-based sealer, and also, there are a large number of dentinal tubules present in the coronal third. If many dentinal tubules are present [15], there will be more resin penetration and resin tag formation. In return, this leads to the higher bond strength of the sealer. The push-out bond strength of AH Plus is considerably higher when compared with MTA Fillapex after rinsing with either normal saline or Chlorhexidine as the only irrigant. The reasons for the higher push-out bond strength of AH Plus might be due to a covalent linkage between an open epoxide ring and exposed amino groups in collagen protein, lower shrinkage while setting, long-term dimensional stability, and inherent volumetric expansion of AH Plus. These factors may contribute to a better AH Plus sealer bond strength [16–18].

## Conclusion

This in vitro study was restricted to single-rooted teeth, using straight root canals, and therefore, the conclusions cannot be directly applied to the teeth with curved root canals. Further in vitro and in vivo studies should be conducted to validate the result of this study with a larger sample size. Our findings showed that among various medicaments, the mean bond strength of the AH Plus sealer was significantly better than the bond strength of the MTA Fillapex sealer (p<0.05). According to the results obtained in this study, it was evident that the adherence of sealer to dentin is dependent on the kind of sealer and intracanal medicaments used. Based on the findings, it thus may be proposed that the AH Plus sealer might be more efficient than other sealers concerning bond strength to root dentin. The other sealer, MTA Fillapex, was seen to have lower adherence to root dentine. Few more studies can be expected to confirm the efficacy of MTA-based sealers with different intracanal medicaments.

## Acknowledgments

### Conflict of interest

The authors declare no conflict of interest.

### Personal thanks

We want to acknowledge the support of Santosh University management.

### Authorship

PS, VDB, and PK contributed to the conceptualization of the research. SM and VDB contributed to the methodology and writing of the manuscript. SM, VDB, and RK contributed to data collection, compilation, analysis, and final editing. Some valuable additions in the discussion part were made by SG, SAN, AG, PK. Revised editing was done by SY. Final approval of the manuscript was done by PK and PS.
